# Characteristics of users of HIV self-testing in Kenya, outcomes, and factors associated with use: results from a population-based HIV impact assessment, 2018

**DOI:** 10.1186/s12889-022-12928-0

**Published:** 2022-04-02

**Authors:** Jonathan Mwangi, Fredrick Miruka, Mary Mugambi, Ahmed Fidhow, Betty Chepkwony, Frankline Kitheka, Evelyn Ngugi, Appolonia Aoko, Catherine Ngugi, Anthony Waruru

**Affiliations:** 1 US Centers for Disease Control and Prevention, Division of Global, HIV & TB, Nairobi, Kenya; 2 Ministry of Health Kenya, National AIDS &, STI Control Program, Nairobi, Kenya; 3 National Public Health Laboratory, Nairobi, Kenya

**Keywords:** HIV testing, HIV self-testing, Population-based HIV Impact Assessment (PHIA), Kenya

## Abstract

**Background and setting:**

About 20% of persons living with HIV aged 15–64 years did not know their HIV status in Kenya, by 2018. Kenya adopted HIV self-testing (HIVST) to help close this gap. We examined the sociodemographic characteristics and outcomes of self-reported users of HIVST as our primary outcome.

**Methods:**

We used data from a 2018 population-based cross-sectional household survey in which we included self-reported sociodemographic and behavioral characteristics and HIV test results. To compare weighted proportions, we used the Rao-Scott χ-square test and Jackknife variance estimation. In addition, we used logistic regression to identify associations of sociodemographic, behavioral, and HIVST utilization.

**Results:**

Of the 23,673 adults who reported having ever tested for HIV, 937 (4.1%) had ever self-tested for HIV. There were regional differences in HIVST, with Nyanza region having the highest prevalence (6.4%), *p* < 0.001. Factors independently associated with having ever self-tested for HIV were secondary education (adjusted odds ratio [aOR], 3.5 [95% (CI): 2.1–5.9]) compared to no primary education, being in the third (aOR, 1.7 [95% CI: 1.2–2.3]), fourth (aOR, 1.6 [95% CI: 1.1–2.2]), or fifth (aOR, 1.8 [95% CI: 1.2–2.7]) wealth quintiles compared to the poorest quintile and having one lifetime sexual partner (aOR, 1.8 [95% CI: 1.0–3.2]) or having ≥ 2 partners (aOR, 2.1 [95% CI: 1.2–3.7]) compared to none. Participants aged ≥ 50 years had lower odds of self-testing (aOR, 0.6 [95% CI: 0.4–1.0]) than those aged 15–19 years.

**Conclusion:**

Kenya has made progress in rolling out HIVST. However, geographic differences and social demographic factors could influence HIVST use. Therefore, more still needs to be done to scale up the use of HIVST among various subpopulations. Using multiple access models could help ensure equity in access to HIVST. In addition, there is need to determine how HIVST use may influence behavior change towardsaccess to prevention and HIV treatment services.

## Introduction

HIV diagnosis through testing is the doorway to HIV prevention and antiretroviral therapy (ART) services [1], whose benefits are well documented [2, 3] and are critical for reducing transmissions and achieving epidemic control [4]. To attain HIV epidemic control, the Joint United Nations Programme on HIV/AIDS (UNAIDS) set 90–90-90 targets: 90% of all people living with HIV (PLHIV) knowing their HIV status; of these, 90% receiving sustained ART; and of these, 90% having viral suppression by 2020 [5]. In 2015, UNAIDS revised these targets to 95–95-95 by 2030 [6]. In addition, the UNAIDS recommended broadening testing options to attain the first target, including community-based testing, home-based self-testing, events, location-based testing, community mobilization for testing, public–private partnerships, and voluntary and provider-initiated counseling. The Kenya Ministry of Health adopted these targets in the 2014/2015–2018/2019 Kenya AIDS Strategic Framework [7].

Even with comprehensive HIV testing strategies and a global increase in the percentage of people living with HIV (PLHIV) who know their HIV-positive status (from 71% in 2015 to 84% in 2020), testing gaps still exist, especially among men and young people. About 16% of PLHIV globally and 10% of adults aged 15 years and older in eastern and southern Africa were unaware of their HIV status in 2020 [8] and about 20% of PLHIV aged 15–64 years were unaware of their HIV status in Kenya in 2018 [9]. Several studies have demonstrated high acceptability and effectiveness of HIVST as a strategy for reaching men and young people [10–13]. In its 2015 guidelines, the World Health Organization (WHO) recommended HIV self-testing as an effective strategy to narrow the gap and increase HIV status knowledge among PLHIV [1]. In 2016, WHO’s HIVST and assisted partner notification services guidelines emphasized HIVST as a strategy to help identify PLHIV [14]. Kenya adopted these WHO guidelines and rolled out HIVST guidelines that included both oral and blood-based HIVST [15]. In Kenya, studies continue to show feasibility and acceptability of HIVST among diverse users in the population [16–19].

Despite studies showing high acceptability for HIVST, few studies have looked at prevalence of HIVST use at the population level [20]. In Zimbabwe and Malawi a population based survey found 1.2% prevalence of use of HIVST [21]. In Kenya, after rolling out HIVST guidelines [15], information on the prevalence of HIVST use and the characteristics of HIVST users is limited. To address this, we used data from a population-based HIV impact assessment survey to characterize HIVST users in Kenya, HIV status outcomes, and factors associated with HIVST use.

## Methods

### Study design and population

The methods used in the 2018 Kenya Population-based HIV Impact Assessment (KENPHIA) 2018 have been previously described . Briefly, KENPHIA (October 2018–February 2019) was a cross-sectional household survey targeting adults aged 15–64 years and children ≤ 14 years old. The survey was a two-stage, stratified cluster sample design with the sampling frame that comprised of all households in the country, based upon the National Sample Survey and Evaluation Program version 5, (NASSEP-V) sampling frame. In the first stage, 800 clusters within the 47 counties of Kenya were selected using a probability proportional to size method. During the second stage, a sample of households was randomly selected within each cluster, using an equal probability method. We restricted our analysis to respondents aged 15–64 years who had ever been tested for HIV.

### Data collection methods

Respondents were interviewed using a standardized PHIA questionnaire regarding household and demographic characteristics, bio-behavioral factors, and use of HIV-related services such as HIV testing services (HTS) and having ever used an HIVST kit. These data were collected on tablet computers and transmitted electronically to a central database. Since receipt of test results was a requirement for participation in the biomarker component, if an individual did not want to receive his or her HIV test result, this was considered a refusal, and the survey was concluded. For respondents consenting to receive test results, HIV home-based counseling and testing were conducted in each household per national guidelines via a sequential rapid-testing algorithm. The first screening test was with Determine HIV 1/2 RT; individuals with a non-reactive test result were reported as HIV negative. No further HIV testing was performed at home. Persons with a reactive result underwent confirmatory testing at home using a second rapid test (First Response HIV 1–2.0 Card Test [Premier Medical Corporation, Mumbai, India]). Those with a reactive result on both screening and confirmatory tests were classified as HIV positive. For quality assurance, whole-blood specimens collected in the household were transported to satellite laboratories. The first 50 tests from each tester and a fraction of negative specimens were tested using the national HIV rapid testing algorithm and confirmatory testing to determine field results’ accuracy. In addition, all HIV-positive specimens were confirmed with the Geenius HIV-1/2 supplemental assay (Bio-Rad Laboratories, Redmond, WA United States).

### Measures

We included the following sociodemographic characteristics for this secondary analysis: sex, residence (urban/rural), age, education, marital status, and wealth quintile. We also included sexual behavioral factors such as sexual encounters in the last 12 months, lifetime sexual partners, and age at sexual debut. We selected the variables due to their relevance in HIVST uptake. Some variables, such as residence and geographic locations, were predetermined from the sampling frame at the survey cluster level. Wealth quintiles were calculated using an established process considering household possessions and income. We categorized the age in years into age bands. Our primary outcome was the prevalence of HIVST use and characteristics associated with HIVST users. The respondents reported their sex, age, education, marital status, and household possessions, and HIVST use during face-to-face interviews. We included the HIV test results by merging the laboratory results with the individual questionnaire response datasets for respondents who consented to a blood draw and testing.

### Analysis

We used PROC SURVEYFREQ in SAS to compare the independence of weighted proportions using the Rao-Scott chi-square statistical test, accounting for the sample design. We used jackknife weights for variance estimation. We tested for associations of sociodemographic, behavioral, and HIV testing services utilization with HIVST and presented both unadjusted and adjusted odds ratios. For the unadjusted logistic regression model, the factors were selected a priori for comparability because they were relevant for the HIV program. In the bivariate analyses, significant covariates at *p* < 0.05 level were then fitted into a multivariable logistic regression model. We additionally assessed for collinearity of factors in the multivariate model and determined that they were not collinear. In all analyses, *p*-values < 0.05 were considered statistically significant.

## Results

**Fig. 1:**
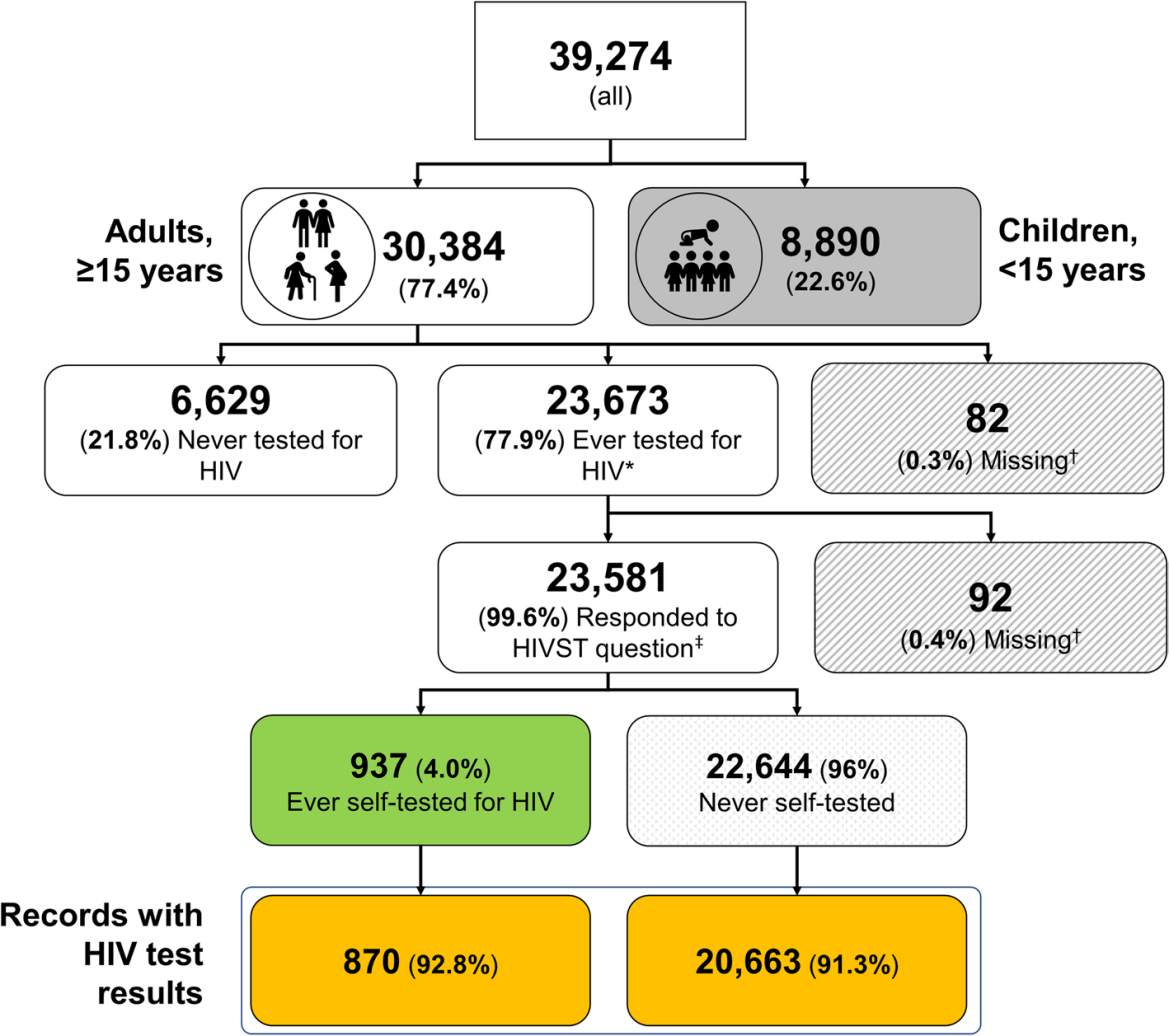
Adolescents and adults reporting to have ever tested for HIV and self-testing, Kenya Population-Based HIV Impact Assessment (KENPHIA 2018). The figure shows how the data were subset for analysis.  The percentages are not weighted. * Self-reported testing; † includes unknown; ‡ HIVST – HIV self-testing

Of the 30,384 2018 KENPHIA participants aged 15–64 years, 23,673 (77.9%) had ever tested for HIV; of these, 23,581 (99.6%) responded to the HIVST question (Fig. 1).

Those who reported to have ever self-tested were 937, 4.0% (95% confidence interval (CI): 3.7–4.6). Most of the respondents who never had self-tested came from urban areas 50.8%, and residents of rural areas had the highest proportion of non-self-testers, 60%, (*p* < 0.001). The older respondents aged ≥ 50 years and younger respondents, 15–19 years had the lowest percentage of self-testers, 7.0%, and 7.3%, respectively, (*p* < 0.001). The highest proportion of self-testers was persons who had secondary education or higher 38.9% (95% CI: 34.3—43.5), *p* < 0.001, or had never been married 50.0% (95% CI: 44.9—55.1), *p* = 0.033, or were wealthiest 31.5% (95% CI: 25.2—37.9), *p* < 0.001, or had sex within the past 12 months 78.9% (95% CI: 74.9–82.4), *p* < 0.001, or respondents who had ≥ two lifetime sexual partners 66.5% (95% CI: 62.2—70.8), *p* < 0.001, and respondents who had their sexual debut at the age 15–19 years 55.4% (95% CI: 50.8—60.0), *p* = 0.022, (Table 1).

**Table 1:** Sociodemographic and behavioral characteristics and self-reported HIV self-testing status among adolescents and adults aged 15–64 years (*N* = 30,384) – who participated in the 2018 Kenya Population-Based HIV Impact Assessment (KENPHIA)

	**Total**			**Ever self-tested**			**Never self-tested**			***P*-value**
**Characteristic**	**n**	**%**	**95% CI**	**n**	**%**	**95% CI**	**n**	**%**	**95% CI**	
**Total**	**23,581**			**937**	**4.0**	**(3.7–4.6)**^a^	**22,644**			
**Sex**										0.082
Male	8945	44.9	(44.5—45.3)	407	48.7	(44.1—53.3)	8538	44.8	(44.3—45.2)	
Female	14,636	55.1	(54.7—55.5)	530	51.3	(46.7—55.9)	14,106	55.2	(54.8—55.7)	
**Residence**										< 0.001
Urban	9322	40.4	(38.4—42.4)	480	50.8	(45.3—56.2)	8842	40.0	(37.9—42.0)	
Rural	14,259	59.6	(57.6—61.6)	457	49.2	(43.8—54.7)	13,802	60.0	(58.0—62.1)	
**Age, years**										< 0.001
15–19	2638	12.3	(11.9—12.7)	68	7.3	(5.2—9.4)	2570	12.5	(12.1—12.9)	
20–24	3493	17.3	(17.0—17.5)	198	25.7	(22.3—29.0)	3295	16.9	(16.6—17.2)	
25–29	3628	17.1	(16.8—17.3)	209	24.6	(21.2—28.0)	3419	16.7	(16.4—17.0)	
30–34	3675	15.0	(14.8—15.2)	153	14.0	(10.9—17.1)	3522	15.0	(14.8—15.3)	
35–39	2749	12.0	(11.8—12.2)	97	9.9	(7.7—12.1)	2652	12.1	(11.8—12.3)	
40–49	4099	15.5	(15.3—15.8)	135	11.4	(9.1—13.8)	3964	15.7	(15.4—16.0)	
50 +	3299	10.9	(10.7—11.1)	77	7.0	(5.1—8.9)	3222	11.1	(10.9—11.3)	
**Education**										< 0.001
No primary	1859	5.4	(4.8—6.0)	41	3.2	(2.1—4.3)	1818	5.5	(4.8—6.1)	
Incomplete Primary	11,147	43.8	(42.6—45.1)	297	27.7	(23.9—31.6)	10,850	44.5	(43.3—45.8)	
Complete Primary	7283	34.5	(33.3—35.6)	286	30.1	(26.6—33.7)	6997	34.6	(33.5—35.8)	
Secondary	3274	16.3	(15.1—17.5)	313	38.9	(34.3—43.5)	2961	15.4	(14.2—16.5)	
**Marital status**										0.033
Never married	5820	43.7	(42.6—44.7)	277	50.0	(44.9—55.1)	5543	43.4	(42.3—44.4)	
Monogamous	5017	37.2	(36.2—38.2)	226	32.7	(27.9—37.5)	4791	37.4	(36.4—38.4)	
Polygamous	340	2.0	(1.7—2.4)	17	2.4	(0.8—3.9)	323	2.0	(1.7—2.4)	
Divorced / separated	1869	11.6	(10.9—12.3)	86	11.3	(8.1—14.5)	1783	11.6	(10.9—12.3)	
Widowed	1122	5.5	(5.1—5.9)	30	3.6	(1.9—5.3)	1092	5.6	(5.2—6.0)	
**Wealth**										< 0.001
Lowest	5348	17.7	(16.2—19.1)	117	9.4	(7.2—11.5)	5231	18.0	(16.6—19.5)	
Second	5130	20.7	(19.5—21.9)	150	15.1	(12.1—18.2)	4980	21.0	(19.8—22.2)	
Middle	5122	21.1	(19.9—22.2)	200	20.9	(17.0—24.7)	4922	21.1	(19.9—22.2)	
Fourth	4684	20.7	(19.0—22.3)	238	23.1	(19.0—27.2)	4446	20.6	(18.9—22.2)	
Highest	3294	19.8	(17.9—21.7)	232	31.5	(25.2—37.9)	3062	19.3	(17.4—21.2)	
**Sex ≤ 12 months**										< 0.001
Yes	16,985	72.8	(71.8—73.7)	734	78.6	(74.9—82.4)	16,251	72.5	(71.6—73.4)	
No	6596	27.2	(26.3—28.2)	203	21.4	(17.6—25.1)	6393	27.5	(26.6—28.4)	
**Lifetime sexual partners**										< 0.001
0 partners	1513	7.8	(7.3—8.3)	31	4.0	(2.2—5.8)	1482	8.0	(7.4—8.5)	
1 partner	8002	32.8	(31.6—33.9)	276	29.5	(25.4—33.7)	7726	32.9	(31.7—34.1)	
2 or more	12,505	59.4	(58.2—60.7)	558	66.5	(62.2—70.8)	11,947	59.1	(57.9—60.4)	
**Age at the first sexual encounter**^b^										0.022
< 15	2737	13.6	(12.9—14.3)	112	13.3	(10.5—16.1)	2625	13.6	(12.9—14.3)	
15–19	12,337	58.3	(57.3—59.3)	500	55.4	(50.8—60.0)	11,837	58.4	(57.4—59.5)	
20–24	4700	22.6	(21.7—23.6)	212	27.4	(22.8—31.9)	4488	22.4	(21.4—23.4)	
25 +	1215	5.5	(5.0—6.0)	44	3.9	(2.6—5.3)	1171	5.6	(5.0—6.1)	

*AbbreviationsCI* Confidence Intervals

^a^row percentage

^b^age in years

**Fig. 2:**
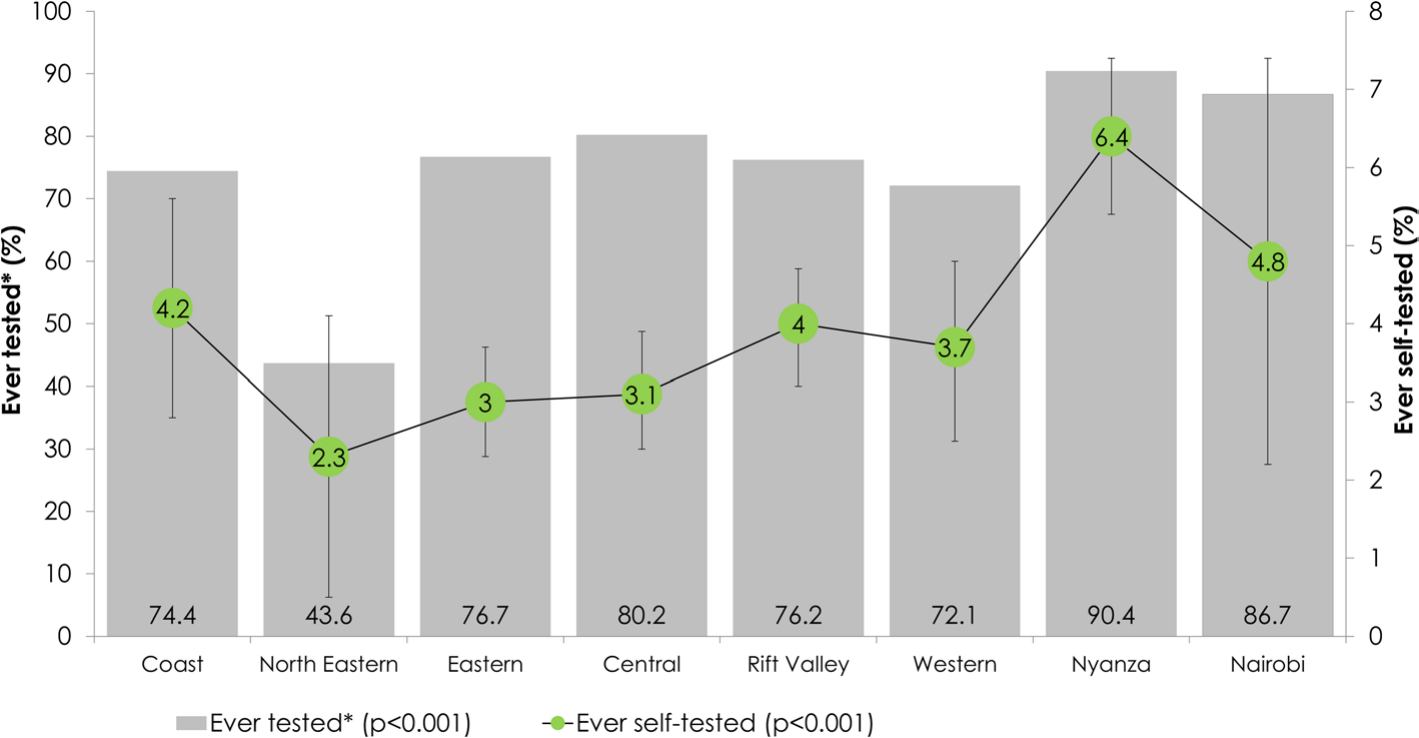
Prevalence of HIV testing and HIV testing across regions, Kenya Population-Based HIV Impact Assessment (KENPHIA 2018). The figure shows regional variation in reported HIV testing and HIV self testing. The percentages are not weighted. * Self-reported testing; † includes unknown; ‡ HIVST – HIV self-testing

Prevalence of HIVST use varied by region, with Nyanza region having the highest prevalence, 6.4%, *p* =  < 0.001 compared to other regions (Fig. 2).

Factors individually associated (unadjusted) with having ever self-tested for HIV were: living in an urban compared to rural setting; being 20–34 years compared to 15–19 years old; completion of primary or secondary education compared to no primary education; having never married compared to being widowed; wealth status in the second to the fifth quintile compared to the lowest quintile; having had sex in the past 12 months compared to none and having one or more partners compared to none. Factors independently (adjusted) associated with having ever self-tested for HIV were secondary education adjusted odds ratio (aOR), 3.5 [95% CI: 2.1–5.9]) compared to no primary education, being in the third (aOR, 1.7 [95% CI: 1.2–2.3]), fourth (aOR, 1.6 [95% CI: 1.1–2.2]), or fifth wealth quintiles (aOR, 1.8 [95% CI: 1.2–2.7]) compared to the first wealth quintile and one-lifetime sexual partner (aOR, 1.8 [95% CI: 1.0–3.2]) or ≥ 2 sexual partners (aOR, 2.1 [95% CI: 1.2–3.7]) compared to those with none (Table 2).

**Table 2:** Factors associated with HIV self-testing among adolescents and adults aged 15–64 years who participated in the 2018 Kenya Population-Based HIV Impact Assessment –(KENPHIA)

**Characteristic**	**Number and percentages**		**Unadjusted** **odds ratios (OR)**		**Adjusted** **odds ratios (aOR)**	
	**Number ever tested for HIV**	**Number and percentage self-tested**	**OR (95% CI)**	***P*-value**	**aOR (95% CI)**	***P*-value**
**Sex**						
Female	14,636	530 (3.8)	ref^a^			
Male	8945	407 (4.5)	1.2 (1.0–1.4)	0.08		
**Residence**						
Urban	14,259	457 (3.4)	ref^a^			
Rural	9322	480 (5.2)	1.6 (1.2–1.9)	< .001	1.0 (0.8–1.3)	0.75
**Age, years**						
15–19	2638	68 (2.5)	ref^a^			
20–24	3493	198 (6.2)	2.6 (1.9–3.6)	< .001	1.3 (0.9–1.9)	0.18
25–29	3628	209 (6.0)	2.5 (1.7–3.6)	< .001	1.2 (0.8–1.9)	0.38
30–34	3675	153 (3.9)	1.6 (1.1–2.3)	0.01	0.9 (0.6–1.4)	0.58
35–39	2749	97 (3.4)	1.4 (0.9–2.1)	0.10	0.7 (0.4–1.2)	0.16
40–49	4099	135 (3.0)	1.2 (0.9–1.8)	0.23	0.8 (0.5–1.2)	0.21
≥ 50	3299	77 (2.7)	1.1 (0.7–1.6)	0.67	0.6 (0.4–1.0)	0.03
**Education**						
No primary	1859	41 (2.5)	ref^a^			
Incomplete Primary	11,147	297 (2.6)	1.5 (1.0–2.2)	0.78	1.1 (0.7–1.9)	0.63
Complete Primary	7283	286 (3.6)	1.5 (1.0–2.2)	0.04	1.4 (0.9–2.4)	0.16
Secondary	3274	313 (9.9)	4.3 (2.9–6.3)	< .001	3.5 (2.1–5.9)	< .001
**Marital status**						
Never married	1122	30 (3.0)	ref^a^			
Monogamous	1869	86 (4.5)	1.5 (0.9–2.6)	0.09		
Polygamous	5017	226 (4.1)	1.4 (0.8–2.3)	0.20		
Divorced/separated	340	17 (5.3)	1.8 (0.8–4.2)	0.14		
Widowed	5820	277 (5.3)	1.8 (1.1–3.0)	0.02		
**Wealth quintiles**						
First (lowest)	5348	117 (2.2)	ref^a^			
Second	5130	150 (3.0)	1.4 (1.1–1.8)	0.02	1.3 (0.9–1.7)	0.1
Third	5122	200 (4.1)	1.9 (1.4–2.6)	< .001	1.7 (1.2–2.3)	< .001
Fourth	4684	238 (4.6)	2.2 (1.6–2.9)	< .001	1.6 (1.1–2.2)	< .001
Fifth (highest)	3294	232 (6.6)	3.1 (2.2–4.5)	< .001	1.8 (1.2–2.7)	< .001
**Sex in the past 12 months**						
No	6596	203 (3.2)	ref^a^			
Yes	16,985	734 (4.5)	1.4 (1.1–1.8)	< 0.001	1.1 (0.8–1.4)	0.54
**Lifetime sexual partners**						
0	1513	31 (2.1)	ref^a^			
1	8002	276 (3.7)	1.8 (1.1–3.0)	0.02	1.8 (1.0–3.2)	0.04
≥ 2	12,505	558 (4.6)	2.2 (1.4–3.6)	< .001	2.1 (1.2–3.7)	0.01

*AbbreviationsCI* Confidence Intervals

^a^referent category

HIV prevalence rates were 4.9% (95% CI: 3.1%–6.7%) among respondents who had ever self-tested for HIV and 6.0% (95% CI: 5.5%–6.4%) among those who never had self-tested for HIV. HIV prevalence varied significantly comparing those who had ever self-tested vs. those who had never self-tested among; persons with incomplete primary education 12.9% vs 8.0% (*p* = 0.015), with secondary education 0.5% vs 2.5% (*p* < 0.001), were never married 0.9% vs 2.6% (*p* = 0.016), were in the lowest wealth quintile 13.4% vs 6.6% (*p* = 0.012), or who had ≥ 2 sexual partners 4% vs 7.7% (*p* = 0.030) (Table 3).

**Table 3:** HIV prevalence by reported HIV self-testing and socio-demographic and behavioral characteristics among adolescents and adults aged 15–64 years (*N* = 21,470) who participated in the 2018 Kenya Population-Based HIV Impact Assessment (KENPHIA)

**Characteristic**	**HIV prevalence**						
	**Ever Self-tested**			**Never Self-tested**			*P*-value*
	**HIV-infected/n**	**%**	**95% CI**	**HIV-infected/n**	**%**	**95% CI**	
**Total**	**50/807**	**4.9**	**(3.1–6.7)**	**1394/20663**	**6.0**	**(5.5–6.4)**	0.265
**Sex**							
Male	18/352	4.4	(1.8–7.1)	383/7719	4.2	(3.6–4.7)	0.821
Female	32/455	5.3	(3.1–7.6)	1011/12944	7.4	(6.8–8.0)	0.100
**Residence**							
Urban	17/401	3.0	(0.9–5.1)	564/7915	5.5	(4.8–6.3)	0.070
Rural	33/406	6.8	(3.9–9.7)	830/12748	6.2	(5.6–6.9)	0.676
**Age, years**							
15–19	0/60	-	-	40/2366	1.5	(0.9–2.1)	†
20–24	7/169	2.0	(0.0–4.1)	80/2980	2.3	(1.7–3.0)	0.780
25–29	10/181	4.0	(1.2–6.8)	163/3081	4.6	(3.7–5.5)	0.672
30–34	12/134	7.2	(2.6–11.8)	252/3201	6.8	(5.8–7.9)	0.866
35–39	2/83	2.8	(0.0–6.8)	192/2416	7.0	(5.7–8.3)	0.169
40–49	9/114	8.2	(2.5–14.0)	378/3628	10.5	(9.2–11.9)	0.473
≥ 50	10/66	17.1	(3.3–30.8)	289/2991	9.6	(8.1–11.1)	0.177
**Education**							
No primary	3/37	8.0	(0.0–17.4)	107/1652	8.8	(6.6–11.1)	0.866
Incomplete Primary	32/260	12.9	(7.9–17.9)	893/10111	8.0	(7.3–8.6)	0.015
Complete Primary	11/252	2.9	(0.9–4.9)	310/6325	4.3	(3.7–4.9)	0.259
Secondary	4/258	0.5	(0.0–0.9)	83/2560	2.5	(1.8–3.3)	< 0.001
**Marital Status**							
Never married	5/231	0.9	(0.0–1.8)	178/4987	2.6	(2.1–3.2)	0.016
Monogamous	14/192	6.1	(2.5– 9.6)	246/4331	5.0	(4.2–5.8)	0.502
Polygamous	2/17	10.7	(0.0–26.0)	28/294	9.3	(5.7–12.9)	0.850
Divorced/separated	11/78	14.0	(2.4–25.7)	174/1643	10.9	(9.1–12.7)	0.557
Widowed	3/23	14.4	(0.0–31.6)	267/1022	28.0	(24.6–31.5)	0.198
**Household Wealth**							
First (lowest)	12/108	13.4	(5.7–21.1)	322/4846	6.6	(5.5–7.6)	0.012
Second	10/128	5.4	(1.7–9.0)	348/4654	6.8	(5.8–7.7)	0.480
Third	14/177	7.1	(2.9–11.2)	336/4543	6.8	(5.7–8.0)	0.910
Fourth	11/202	3.1	(0.7–5.4)	262/3996	5.4	(4.5–6.3)	0.123
Fifth (highest)	3/192	2.0	(0.0–4.9)	125/2622	4.0	(3.0–5.0)	0.323
**Lifetime sexual partners**							
0	0/22	-	-	24/1314	1.9	(0.9–2.9)	†
1	10/238	3.2	(0.5–5.9)	266/6956	3.6	(2.9–4.2)	0.779
≥ 2	33/485	5.0	(2.9–7.1)	1015/11069	7.7	(7.0–8.3)	0.030
**Age at first sex, years**							
< 15	12/99	8.6	(2.6–14.7)	228/2454	7.6	(6.3–8.8)	0.706
15–19	29/446	5.9	(3.3–8.5)	812/10896	6.5	(5.9–7.2)	0.626
20–24	6/179	2.0	(0.0–4.1)	220/4037	4.9	(4.1–5.8)	0.062
≥ 25	1/35	1.7	(0.0–4.2)	47/1045	4.7	(2.9–6.5)	0.147

*Abbreviations*: *CI* Confidence Interval

^*^Rao-Scott χ-square statistical test *p*-values are computed for each of the categories as two-by-two tables of ever having self-tested, and the outcome is HIV prevalence

^†^*p*-value not calculated due to missing values

## Discussion

Among the survey respondents who reported having had an HIV test, we found that 4.0% reported having ever taken an HIV self-test. Comparatively, among those who had had an HIV test in Malawi and Zimbabwe, 1.0% and 1.2%, respectively, reported having ever taken an HIV self-test in a population based survey [21]. The results also showed geographic variation in the prevalence of HIVST use. This geographic variation largely mirrors HIV prevalence in the country and the corresponding efforts to increase access to HIV prevention and treatment services in Kenya. The relatively low prevalence of HIVST provides an opportunity to scale up the use of HIVST kits to meet the demand for HIVST among various populations, as has been demonstrated in previous studies. For example, in a prior survey in Kenya, 70% of the respondents reported willingness to use HIVST privately or at home (men, 74%; women, 67%) [22]. Similarly, other studies have reported high acceptability rates of HIVST among the general population [23, 24] and key populations [25]. To increase access to HIVST, the Ministry of health in Kenya developed the HIVST guidelines [15], informed by multiple studies on HIVST acceptability and impact to reach populations [26, 27].

Among those reporting to have ever used an HIV self-test, we found that participants aged 20–29 years were more likely to use HIVST kits, and those older than 50 years were less likely to self-test. A study in Malawi found a similar pattern of decreasing the use of HIVST across older age groups. This was attributed to possibly frequent access to health facilities by the younger population, where HIVST are distributed [28]. These findings could help inform Kenya’s HIV testing program strategies, whose current HIVST objective is to target partners of pregnant and breastfeeding women, men and young persons to close the gaps in the knowledge of HIV status among these groups [22]. However, even though these target populations have a relatively higher prevalence of HIVST use, further scale-up is still needed to expand the prevalence of HIVST use across all age groups. A large-scale rollout of HIVST with different approaches has been practiced in Malawi, Zambia, and Zimbabwe [12]. Similarly, Kenya’s HIVST guidelines provide multiple distribution channels that include facility-based, community-based, and private-sector channels that utilize pharmacies where individuals can buy self-testing kits [15] at approximately five US Dollars [29]. At health facilities, and private pharmacies, there is an option of utilizing the HIVST under the guidance of a healthcare worker (assisted HIVST).

Higher wealth quintiles were associated with higher HIVST prevalence, possibly because of the higher purchasing power among those respondents [30]. This finding suggests possible inequity in access to HIVST. Furthermore, in this survey, those in the lowest quintile reported a higher prevalence of HIV but reported the most insufficient use of HIVST. This finding underlines the need to ensure all populations are reached, irrespective of socioeconomic status. Demand for HIVST is price-sensitive [31, 32], and price may create inequalities to access where the pricing is considered out of reach to segments of the population. A mix of methods [33, 34], including free distribution of HIV self-tests [31], secondary distribution [26], use of vouchers [35], text message reminders [36], and internet-based approaches [37], may help promote access and use in targeted populations.

We also found higher use of HIVST by those with two or more lifetime sexual partners. This could be associated with participants’ perception of their susceptibility to infection [38]. Individuals with multiple sexual partners are at higher risk of HIV infection [39, 40] and perceived susceptibility has been described as a predictor of HIVST use [41]. Moreover, in this survey, among individuals with ≥ two lifetime sexual partners, those who reported having self-tested for HIV had a lower prevalence of HIV compared to those who had never been tested. This finding warrants further investigation to determine how use of HIVST may influence behavior change towards access of HIV prevention and treatment services.

Although HIVST offers a convenient approach to knowing one’s HIV status, linkage to treatment and other prevention services remains a challenge to be addressed [42], considering privacy and confidentiality is a key advantage of HIVST. Financial incentives [43] and interactive voice response systems [44] have demonstrated potential in increasing the linkage to HIV treatment services. Monitoring ART enrollment and population-based surveys have been proposed for programs to monitor linkage to treatment from HIVST [45]. More research is warranted to explore ways of increasing access to HIVST and linkage to prevention and treatment services among all populations.

### Study strengths and limitations

The study had a large sample size from a survey distributed across the country, thus providing a nationally representative sample.

Our findings are subject to several limitations. First, the HIVST question posed during the survey may have been subject to social-desirability bias in responses like all questions asked in face-to-face interviews. However, the HIVST prevalence is comparable to others reported elsewhere in similar PHIA surveys. Second, the KENPHIA survey was not powered to characterize HIVST use in smaller geographical regions but provided national estimates.

## Conclusions

From the survey, among those who reported having ever tested for HIV, 4.0% reported having ever self-tested for HIV. Those living in urban areas had a higher prevalence of HIVST use compared to those living in rural areas. Younger age, higher education levels, being of higher wealth quintile, and having multiple lifetime sexual partners were associated with the use of HIVST. While progress has been made by the program in Kenya to roll out HIVST, more may still need to be done to scale up the use of HIVST among various subpopulations and these results could serve as a baseline. The Kenya program could explore using multiple access models to help ensure equity in access to HIVST. In addition, there is a need to determine the impact of HIVST on behavior change towards access to prevention and HIV treatment services.

## Data Availability

The datasets used and analyzed during the current study are available from the corresponding author on reasonable request.

## Abbreviations

AIDS: Acquired Immunodeficiency syndrome
aOR: Adjusted odds ratio
ART: Antiretroviral therapy
CI: Confidence internal
HIV: HIV: Human immunodeficiency virus
HIVST: HIV self-testing
HTS: HIV Testing services
KENPHIA: Kenya Population-based HIV Impact Assessment
NASSEP-V: National Sample Survey and Evaluation Program version 5
PHIA: Population-based HIV Impact Assessment
PLHIV: People Living with HIV
TB: Tuberculosis
UNAIDS: The Joint United Nations Programme on HIV/AIDS
US: United States of America
WHO: World health organization
